# Evaluation of Immunization Coverage in the Rural Area of Peshawar, Khyber Pakhtunkhwa

**DOI:** 10.7759/cureus.3992

**Published:** 2019-01-31

**Authors:** Adnan Khan, Sarbiland Khan, Irfan Ullah, Saima Yaseen, Ghulam H Khan, Hina Rashid, Humera Jamil, Tehrim Tahir, Ayesha Riaz

**Affiliations:** 1 Pediatrics, Rehman Medical Institute, Peshawar, PAK; 2 Internal Medicine, Rehman Medical Institute, Peshawar, PAK; 3 Internal Medicine, Medical Teaching Institute, Bannu, PAK; 4 Internal Medicine, Norfolk & Norwich University Hospital, Norwich, GBR; 5 Obstetrics and Gynecology, Rehman Medical Institute, Peshawar, PAK; 6 Epidemiology and Public Health, Rehman Medical Institute, Peshawar, PAK

**Keywords:** polio, immunization, 30-cluster sampling

## Abstract

Background: In children, the leading cause of morbidity and mortality is infectious disease. Immunization is one of the most cost-effective methods for child survival. The purpose of the survey is to assess access and coverage of immunizations in the rural areas of the District Peshawar, Khyber Pakhtunkhwa.

Methods: A cross‑sectional study was conducted in a rural population area of District Peshawar from February 2016 to April 2016 using the WHO’s 30 cluster sampling method for evaluation of immunization coverage.

Results: A total of 390 children aged 12-23 months were included in the study. It was found that 67.94% of the children were fully immunized against vaccine-preventable diseases. Thirty percent of the children were partially immunized; the percentage of unimmunized children was 2.06%. Immunization cards were issued to and available with 58.8% of the subjects. The most common cause of partial immunization was a lack of information regarding vaccinations (27%). Immunization against measles was found to be low (67%). Those using private facilities were more likely to be completely immunized as compared to government facilities.

Conclusions: Immunization coverage in our survey was 68%. Sustained efforts are required to achieve universal coverage of immunization. Significant interventions are required, especially in areas that are more rural and less educated.

## Introduction

In children, the major cause of morbidity and mortality is infectious disease. The vaccine is one of the most considerable augmentations to the field of medicine; immunizations are one of the most cost-effective methods for child survival. They are the most efficacious, low-priced, and invulnerable means for eradication of a variety of illnesses.

Smallpox elimination was one of the historical accomplishments that alerted and induced a prompt Disease Early Warning System extensive and indiscriminate field response against the vaccine-preventable infectious diseases by World Health Organization (WHO) and to redress the prevailing issue along with its affiliated countries to launch a global immunization campaign in contrast to six vaccine-preventable diseases in its national immunization program. The WHO initiated an Expanded Programme on Immunization (EPI) worldwide in May 1974, with its focal point on hindrance of six vaccine-preventable diseases by the year 2000 [1].

In the USA, the majority of vaccine-preventable diseases are at or almost at the zero level [2]. Immunization by WHO is considered as the most important arbitration for the encouragement of child’s health [3]. Immunizing a child notably decreases the expenditure spent to manage a disease. As a result, it imparts a beneficial incentive towards alleviation of poverty.

In Pakistan, EPI was launched in 1978, with support from WHO and UNICEF. There was a marked achievement in access and coverage from 20% in 1980 to over 80% in 1996 in the portion of world’s children immunized against the major vaccine-preventable diseases. This averted more than 2.8 million children deaths per year [4]. Most vaccine-preventable disease remains prevalent even with the success of EPI in the developing world; this has been linked with 20%-35% of all death in children under the age of five [5].

Immunization broadcasting needs improvement in Pakistan. Knowledge, attitude, and practices (KAP) of parents are noted to contribute to either attainment or decline of immunization curriculum [6].

A KAP study that was carried in the urban population of Northern areas of Pakistan illustrated divergence between knowledge and practice. According to this study, 88% of parents were familiar with the EPI program; 77% of mothers anticipated to vaccination to be beneficial; more than 90% showed positive response and were ready to contribute, but just 71% of them had immunized their children. According to them reasons for not vaccinating their children were parent’s laziness, non-cooperative husbands, and poor quality of service [7].

This study was conducted to assess the coverage of immunization in the rural areas of the Peshawar, Khyber Pakhtunkhwa.

## Materials and methods

A cross-sectional study was carried out in Nahaqi town, District Peshawar from February 2016 to April 2016. The total population of Nahaqi town is 36,431. A total of 390 households with children in the age group 12-23 months on the date of interview were included in the study using WHO 30-cluster survey methodology. The study sample size was calculated by estimating the difference between population proportions using the software, “Sample size determination in health studies” of the WHO. To calculate the sample size, the derived confidence level was taken to be 95% and a confidence interval of 5. A routine coverage of 50% was assumed. Total sample size was 380 rounded to 390 to take care of the situation where some clusters are not accessible. With 30 clusters to be studied, the number of respondents selected per cluster = 390/30 = 13. The cluster interval of 1214 was obtained by dividing the total population by 30. 36,431/30= 1214 (cluster interval). A random number less than the cluster interval was generated with the help of a page of a blindly opened book, that is, the page number was 96. The first cluster having the cumulative frequency equal to or more than 96, was picked as the first cluster interval that is 96+1214 = 1310. Cumulative frequency equal to or more than 1310 was the second cluster. Some 30 clusters were selected. The first household was selected randomly and the next was studied in sequence.

The mother was considered the primary respondent. If the mother was absent, the father acted as the respondent. Background questions covered demographic, social, and economic status. The child was considered as completely immunized if he/she received a single dose of bacille Calmette-Guerin (BCG), three doses of diphtheria, pertussis (whooping cough), and tetanus (DPT), three doses of oral polio virus (OPV) and a single dose of measles, and as unimmunized if he/she had received none of these and partially immunized if some doses were given, but not complete.

The Ethical Review Board of Rehman Medical College, Peshawar, Pakistan approved the study on Feb 2016. (NEH/01/02/16)

Statistical analysis

Statistical analysis was performed using the statistical package for social science (SPSS) version 22. Continuous data were displayed as the mean ± standard deviation (SD). Discrete data was analyzed using Pearson’s Chi-Square test for normal distribution. A p-value, <0.05 was considered significant.

## Results

 A total of 390 children in the age group 12-23 months, of which 214(54.9%) were males and 176(45.1%) were females. The vast majority of mothers were housewives 375(96.1%) and 318(81.4%) had no primary education. The age range for mothers was 20-40 years with a mean of 24.80 ± 4.29 and the age range for fathers was 21-40 years with a mean age of 31.29 ± 4.19 (Table 1).

**Table 1:** Distribution of demographic indicators of the parents of respondents.

Sociodemographic indicator	Father (n = 390)	Mother (n = 390)
1. Age in years		
<20	0	103(26.5%)
21-25	46(11.8%)	165(42.2%)
26-30	191(49.0%)	107(27.5%)
31-35	114(29.4%)	11(2.9%)
36-40	39(9.8%)	4(1%)
>40	0	0
2. Education		
Illiterate	164(42.2%)	318(81.4%)
Primary school	100(25.5%)	57(14.7%)
Secondary school	92(23.5%)	11(2.9%)
Pre-college	23(5.9%)	4(1%)
Graduate	11(2.9%)	0
3. Occupation		
Unemployed/housewife	19(4.9%)	375(96.1%)
Daily wage	256(65.7%)	4(1%)
Employed	115(29.4%)	11(2.9%)

Some 229(58.8%) respondents had the immunization card with them. Most of the children 367(94.1%) received their immunization from the government while 15(3.9%) received it from private setup and 8(2%) did not receive any vaccination. Out of 390 children 265(67.94%) were fully immunized, 117(30%) were partially immunized, and 8(2.05%) were not immunized (Table 2).

**Table 2:** Immunization details of children aged 12-23 months.

Immunization details	Number of children (n = 390)
1. Sex of child	
Male	214(54.9%)
Female	176(45.1%)
2. Immunization card	
Has card	229(58.8%)
Does not have card	161(41.2%)
3. Site of immunization	
Government	367(94.1%)
Private	15(3.9%)
Not given	08(2%)
4. Immunization status	
Fully immunized	265(67.94%)
Partially immunized	117(30%)
Unimmunized	8(2.06%)

Out of 390, 291(74.5%) had knowledge about vaccination in which 364(93.4%) read about vaccination on media and rest were informed by health workers, relatives, and friends while rest of 99(25.5%) had no knowledge.

The majority of the respondents [344(88.2%)] opined that vaccination prevents diseases and only 46(11.8%) said that vaccination does not prevent diseases. 

Some 344(88.2%) were in favor for vaccination and 46(11.79%) were not in favor for vaccination. 

**Figure 1:**
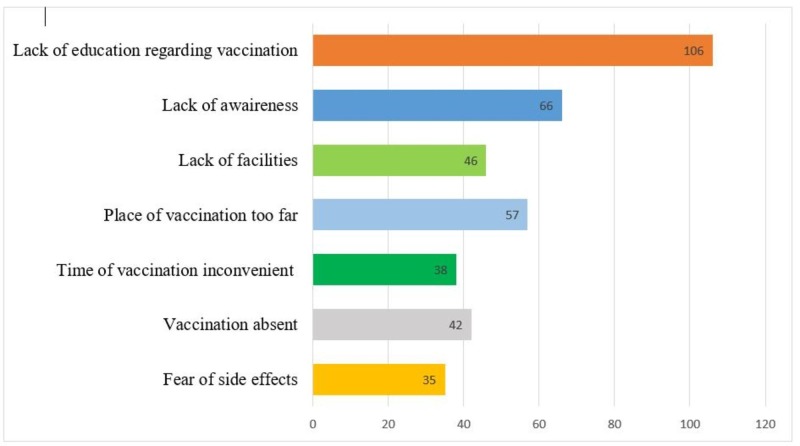
Reason for failure of immunization among the children aged 12-23 months.

Out of 390 majority of the respondents, 360(92.2%) said that vaccination is not harmful while 30(7.8%) said that it is harmful. Some 168(43.1%) thought that vaccination is for all ages while 222(56.9%) said it is only for children. Only 337(86.3%) said that they will recommend vaccination to others whereas 53(13.7%) said that they would not recommend vaccination. By asking about the sources that gave them information about the harmful effects of immunization, 165(42.2%) got information from neighbors, 35(8.8%) from parents, 30(7.8%) from friends, and 4(1%) from the local clerk (mullah). On asking about side effects of immunization 76(19.6%) responded that it is not good for health of the child, 15(14.7%) responded that it caused infertility in children, 38(9.8%) responded that it caused fever and rash, and 214(54.9%) responded that there is no side effect of immunization. Reason for failure of immunization among the children aged 12-23 months is shown in Figure 1.

**Figure 2:**
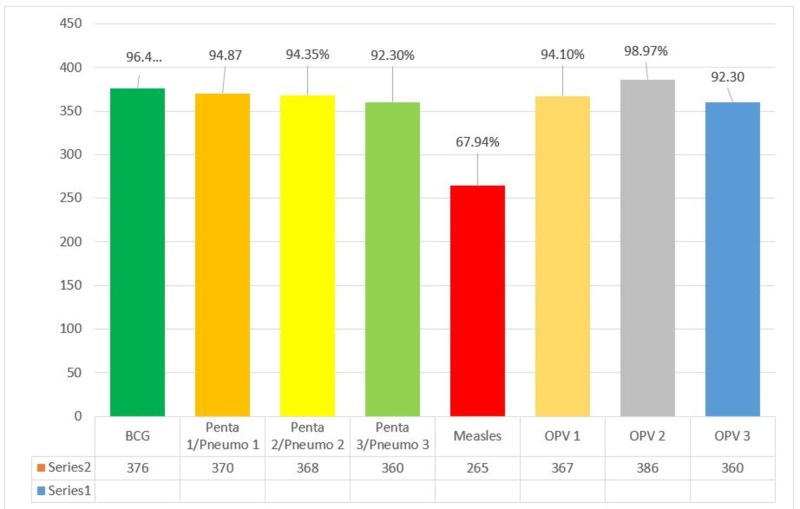
Number of children aged 12-23 months vaccinated with the individual vaccine.

Number of children aged 12-23 months vaccinated with the individual vaccine is shown in Figure 2.

On applying the Chi-Square test, it was found that the effect of factors, such as mother’s educational status, sex of the child and site of immunization on the immunization status, was not statistically significant on mother education (p = 0.787) but it was significant for the gender of the child (p = 0.042) and the site of immunization (p = 0.001) (Table 3).

**Table 3:** Comparison of immunization status with demographic indicators.

	Immunization status		
	Fully immunized	Partially immunized	Unimmunized
1. Mother education			
No education	207	102	8
Primary school	43	15	0
Secondary school	11	0	00
Pre-college	4	0	0
Degree of freedom = 6, Chi Square test = 3.169, p = 0.787			
2. Gender of child			
Male	163	51	0
Female	102	66	8
Degree of freedom = 2, Chi Square = 6.344, p = 0.042			
3. Site of immunization			
Government	250	117	0
Private	15	0	0
Not done	0	0	8
Degree of freedom = 4, Chi Square = 104.496, p = <0.001			

## Discussion

In our study, the percentage of fully immunized children coverage is 67.94%. This is better than the reported Pakistan Demographic and Health Survey done in 2012-2013 which was 54% [8]. This high coverage could be due to better health service in 2016 as compared to 2013; however, there is high variability in the different union councils in the present study. Some union councils had coverage of more than 75% while some had as low as 47%.

The major finding in our study was the lack of education regarding vaccination. Moreover, doubt in the mind of mothers about the nonavailability of vaccine or absence of vaccinator at the place of immunization was also an important factor in the failure of immunization. In our study, only 58.8% could produce the immunization card. This demonstrates that the immunization card may not be considered an important document. One cannot say how reliable the respondent recall is.

The majority (94.1%) of the children received their immunizations from the government; only 3.9% received it privately. Children who received immunizations privately were fully immunized; only 61.4% of those who received immunizations from the government were fully immunized. In our findings, the reason behind the partially and nonimmunized children was the nonavailability of the vaccines at the EPI centers. Measles vaccine was one of them, due to which measles epidemic occurs each year in children below two years. The other reason reported was that the EPI teams’ visit timings were inappropriate; there was also no accurate timing for a visit.

The EPI Department has to improve its performance at health centers and ensure that vaccines are available with proper storage and maintenance of cold chain. In our study, the male to female ratio was 1.2:1. For male children, the majority were fully immunized and some were partially immunized. On the other hand, in female children, almost half were partially immunized. Furthermore, all of the unimmunized children were females. It was also seen that immunization statuses of the children were significantly associated with their genders with the p-value of 0.042. It shows us that gender bias was present due to which most of the females were left partially immunized or unimmunized. This was similar to the study done, in which the coverage of vaccines was higher in males as compared to females; this difference was statistically significant [9].

A majority (74%) of respondents according to our study were informed about vaccination due to the role of media and health workers. It was found that media played a key role in spreading information about immunization as a result of our study.

One of the most important sources in spreading the knowledge regarding immunization is media. This needs to be further improved. Television can be the best source for educating people about immunization. Previous reports have also highlighted the important role of media in encouragement of immunization [10].

It has been reported from India and Bangladesh where nurses and other paramedical workers were mentioned as the main sources of information about immunization for women [11-12]. Similar findings were seen in study, which found that health workers and health personnel were the major sources of information regarding immunization [11]. 

A majority of mothers were in favor of vaccination, that is, every child should be vaccinated. Only 11.8 % were not in favor of vaccination. Some 42% of fathers and 81% of mothers were illiterate in our study. In this matter, the population needs to be educated. For this reason, proper seminars and workshops should be conducted in remote areas by Public Health in order to educate parents. Women should be given special education regarding vaccination by health workers.

Our study reported that 88.2% of mothers have knowledge about vaccine-preventable diseases; similar findings have been reported from studies conducted in India [13]. This area also requires further strengthening. 

In our study, with respect to who provided information about the side effects of immunization, neighbors were on the top. This was followed by parents and friends rather than physicians. The possible side effects from immunization, as well as its adverse impact on immunization coverage have been reported previously [14]. In our study, only 1% source of the side effect was from the local religious cleric (mullah). It is a common perception in the media that in villages and in the remote areas mullahs are responsible for giving wrong information regarding immunization but our study findings showed that the people who live around are the bigger source of giving wrong information. 

In our study, with respect to the polio vaccine: 19.6% said that vaccination is not good for child’s health; 14.7% stated that it causes infertility; and 9.8% said that it causes fever and rash. 

The immunization coverage against diseases such as measles was reported by respondents to be very low for their target children population. This emphasizes an alarming condition that needs to be controlled through urgent measures. High levels of dropout rates were another reason for concern. High levels of initial vaccination rates and low levels of OPV3/DPT3 and measles vaccines were also seen in a study from India [13].

This is a clear indication that the programme needs to focus not only on initiating immunizations, but that it should also concentrate on motivating parents to complete the immunization schedule for their children.

## Conclusions

Immunization coverage of 12-23 months’ children in the field practice area of Rural Peshawar was found to be 68%. Sustained efforts are required to achieve universal coverage of immunization. Educational interventions are needed to upgrade parents’ education with special attention on less edified and destitute inhabitants of rural area. An unfortunate fact was that though, majority of the population recognized the importance of immunization, a superficial cognizance of the schedule and failure of the ascendant entities in inculcating enough motivation in the target population for completing the schedule, has led to a sizably voluminous proportion of the children being partially immunized. Comprehensive strategy should be developed by health authorities, to bring out and achieve effective changes in the rituals and practices regarding immunization of children.
